# Endophytic bacteria with allelopathic potential regulate gene expression and metabolite production in host *Casuarina equisetifolia*

**DOI:** 10.3389/fpls.2024.1435440

**Published:** 2024-09-18

**Authors:** Ying Wang, Pan Chen, Qi Lin, Linzhi Zuo, Lei Li

**Affiliations:** Ministry of Education Key Laboratory for Ecology of Tropical Islands, Key Laboratory of Tropical Animal and Plant Ecology of Hainan Province, College of Life Sciences, Hainan Normal University, Haikou, China

**Keywords:** Casuarina equisetifolia, Bacillus amyloliquefaciens, Bacillus aryabhattai, allelopathy, metabolite

## Abstract

**Introduction:**

*Casuarina equisetifolia* is a common protective forest in coastal areas. However, artificial *C. equisetifolia* forests cannot self-renew, mainly due to the accumulation of allelochemicals. Endophytic bacteria may alleviate the root growth inhibition caused by allelochemicals in *C. equisetifolia* seedlings. *B. amyloliquefaciens* and *B. aryabhattai* were endophytic bacteria with strong allelopathy in *C. equisetifolia* root. The allelopathy mechanism of these two endophytes and their interaction with *C. equisetifolia* remains to be studied.

**Methods:**

Whole-genome sequencing of *B. amyloliquefaciens* and *B. aryabhattai* isolated from the roots of allelochemical-accumulating *C. equisetifolia* was performed using Illumina Hiseq and PacBio single-molecule sequencing platforms. Sterile seedlings of *C. equisetifolia* were treated with either individual or mixed bacterial cultures through root drenching. Transcriptional and metabolomics analyses were conducted after 3 days of infection.

**Results and discussion:**

Whole-genome sequencing of *Bacillus aryabhattai* and *Bacillus amyloliquefaciens* showed that the two strains contained various horizontal gene transfer elements such as insertion sequence, prophage and transposon. In addition, these two strains also contain numerous genes related to the synthesis and catabolism of allelochemicals. After these two strains of bacteria were individually or mixed infected with *C. equisetifolia*, metabolomics and transcriptomic analysis of *C. equisetifolia* showed the 11 important secondary metabolite biosynthesis among them alkaloids biosynthesis, phenylpropanoid and terpenes biosynthesis and related genes were putatively regulated. Correlation analysis revealed that 48 differentially expressed genes had strong positive correlations with 42 differential metabolites, and 48 differentially expressed genes had strong negative correlations with 36 differential metabolites. For example, CMBL gene showed positive correlations with the allelochemical (-)-Catechin gallate, while Bp10 gene showed negative correlations with (-)-Catechin gallate.

**Conclusion:**

The intergenerational accumulation of allelochemicals may induce horizontal gene transfer in endogenic bacteria of *Casuarina equisetifolia* root. Endophytic *Bacillus* plays an allelopathic role by assisting the host in regulating gene expression and the production and/or variety of allelochemicals. This comprehensive study sheds light on the intricate genetic and metabolic interactions between *Bacillus* endophytes and *C. equisetifolia*. These findings provide insights into endophyte-mediated allelopathy and its potential uses in plant biology and forest sustainability.

## 1 Introduction

Allelopathy, the biological phenomenon in which plants send biochemical (allelochemicals) into the environment to impact the growth, survival, and reproduction of surrounding plants, is an important ecological and evolutionary process (Xiu-Feng and Yang, 2007; Yang et al., 2018; Mushtaq et al., 2020b; Ain and Mushtaq, 2023; Mushtaq et al., 2024)*. Casuarina equisetifolia* is an evergreen tree belonging to the *Casuarinaceae* family and *Casuarina* genus (Ahmad et al., 2022). This tree is frequently used in protection forests in coastal areas (Wei et al., 2021) and is instrumental in maintaining the stability of coastal ecosystems, safeguarding coastal agriculture, aiding in wind prevention and sand fixation, dike protection, and tsunami resistance (Zhong et al., 2010). However, with increasing age, *C. equisetifolia* forests may face degeneration and challenges in self-renewal (Xu et al., 2022), resulting in reduced protective efficacy. These issues are likely attributed to decreased microbial activity and nutrient deficiencies. *C. equisetifolia* is a symbiotic plant that relies on several endophytic bacteria for nutrition (Lin et al., 2022b). Its allelopathic potential is closely linked to its complex interactions with its microbiome, particularly with endophytic microbes (Mushtaq and Siddiqui, 2018; Huang et al., 2020). The main reason for this reduction may be the accumulation of allelochemicals released by the plant’s root system. In addition, the allelopathic influence of Casuarina is known to affect seed germination, seedling growth, and overall plant health (Ahmed et al., 2019). For instance, compounds such as juglone, which is a naphthoquinone, have been shown to inhibit root and shoot growth in some plant species (Medic et al., 2021). Additionally, Casuarina’s allelopathic effects can alter soil microbial communities, which may further impact plant growth and soil health (Batish et al., 2001).

Endophytes are microorganisms that live within plant tissues for a significant portion of their life cycle without often producing unwanted consequences to the plants (Compant and Cambon, 2021). They are mainly known for their beneficial involvement in their host plant, and perform critical roles in improving plant fitness and resilience (Fagorzi and Mengoni, 2022). These symbiotic partners have a variety of roles in plant biology, including growth stimulation, disease resistance, and abiotic stress tolerance (Singh et al., 2011). Endophytes frequently develop multicellular aggregation communities in plants, which have gained attention as a unique “microecological” relationship between endophytes and host plants (Jia et al., 2016; Bziuk and Maccario, 2021; Delaux and Schornack, 2021). In recent years, there has been a growing interest in understanding how endophytes affect the allelopathic behavior of their host plants. Endophytes and their hosts have a complex relationship that includes gene regulation and secondary metabolite synthesis, both of which can mediate allelopathic interactions (Andersen et al., 2013; Alam et al., 2021). Previous research has shown that *B. amyloliquefaciens* can produce antibiotics and lipopeptides that inhibit the growth of phytopathogens, thereby enhancing the overall health and resistance of the host plant (Yang et al., 2024). Similarly, both *B. amyloliquefaciens* and *B. aryabhattai* have been found to induce systemic resistance in plants through the activation of defense-related pathways such as the salicylic acid and jasmonic acid pathways (Yu et al., 2022). In addition, is has been shown that the endophytic bacteria are known to produce phytohormones like indole-3-acetic acid (IAA), which promote root growth and development, thereby indirectly influencing root metabolism and overall plant vigor (Adeleke et al., 2021). By the inoculation of *B. amyloliquefaciens* and *B. aryabhattai* into the roots of *C. equisetifolia*, we aim to learn more about the allelopathic mechanisms mediated by endophytic bacteria.

Allelochemicals are almost all secondary metabolites of plants or microorganisms, mainly including organic acids, ketones, terpenes, phenols, alkaloids, glycosides, amino acids and peptides (Kong et al., 2019; Macías et al., 2019). Secondary metabolites are the byproducts of plant growth and development that interact with the surrounding environment. Furthermore, microbes can reduce allelopathic effects by degrading Secondary metabolites, allowing target plants to be more resistant to them (Liu et al., 2018; Bonanomi et al., 2021). They can also remove insoluble phytotoxins linked to intolerant components and convert innocuous molecules into phytotoxins to aggravate the allelopathic effects (Barto et al., 2011; Cipollini et al., 2012). Similarly, numerous research has demonstrated the allelopathic potential of *Casuarina equisetifolia*, emphasizing the presence of allelochemicals such as phenolic compounds, flavonoids, and terpenoids (Li et al., 2010; Tian et al., 2017). These secondary metabolites are essential for both competitive strategies and plant defense (Divekar and Narayana, 2022). On the other hand, the synthesis of these compounds and control are sophisticated processes involving complex gene networks and signaling pathways (Amiri and Moghadam, 2023). However, secondary metabolites are vital components that plants use to defend themselves and adapt to their surroundings. They perform a variety of physiological tasks, including controlling plant growth and biological (Fernie and Pichersky, 2015; Erb and Kliebenstein, 2020). Previous research demonstrated that a variety of environmental conditions, including light, temperature, and microbes, have an impact on the synthesis of different secondary metabolites in plants (Xiu-Feng and Yang, 2007). In addition, it has been reported that endophytes are essential for regulating these pathways and increasing the allelopathic activity in plants (Li et al., 2023).

Our previous research identified the endophytes involved in the accumulation of allelochemicals within *C. equisetifolia* roots (Lin et al., 2022a). Specifically, *Bacillus amyloliquefaciens* and *B. aryabhattai*, isolated from *C. equisetifolia* roots, exhibited allelopathic effect indices of -1 and -0.99, respectively (Huang, 2019). These were the most potent allelopathic strains among all endophytic bacteria discovered in *C. equisetifolia* roots. These strains produce metabolites such as 2,2′-methylenebis(6-tert-butyl-4-methylphenol), 1,2,3,4-butanetetrol, 4-methoxy-3,5-dihydroxybenzoic acid, 3,4,5-trihydroxybenzoic acid, 3-aminophenol, 3,5-dimethyoxy phenyl hydroxide, and p-phenylene diamine, all of which are recognized allelochemicals (Mushtaq et al., 2020a; Zhang et al., 2020). Earlier studies on *B. amyloliquefaciens* and *B. aryabhattai* primarily focused on their role in promoting plant growth (Soares et al., 2016; Bhattacharyya et al., 2017). However, many questions remain about how these bacteria interact with their plant hosts to produce secondary metabolites involved in allelopathy, which genes at the molecular level are responsible for synthesizing these compounds, and how the presence of these bacterial strains influences the production of specific secondary metabolites. Gaining insights into the interactions between these bacteria and C. equisetifolia could enhance the plant’s growth and health, offering significant benefits for forestry and agriculture.

The study aims to understand how these endophytic bacteria influence allelopathy in *C. equisetifolia* through changes in gene expression and metabolite production. The combines Illumina Hiseq and PacBio single-molecule sequencing technologies that were performed for whole-genome sequencing of *B. amyloliquefaciens* and *B. aryabhattai* isolated from *C. equisetifolia* roots with accumulated allelochemicals. The first objective was to obtain genomic information from these strains to deepen our understanding of their allelopathic mechanisms from a genetic perspective and better understand the involvement of endophytes in *C. equisetifolia* allelopathy. The second objective is to comprehend how these endophytes regulate the host plant’s gene expression and secondary metabolite production to enhance allelopathic interactions. Lastly, we aim to identify the genes associated with allelochemical synthesis and the differential metabolites through transcriptomics and non-targeted metabolomics analyses. By a more comprehensive understanding of how endophytic bacteria regulate allelopathy and tree growth, the study may lead to more environmentally friendly forestry practices for species like *C. equisetifolia*, which has been used for timber, erosion control, and land reclamation.

## 2 Results

### 2.1 Characteristics of the genome composition of endophytic bacilli from *C. equisetifolia*

**Figure 1:**
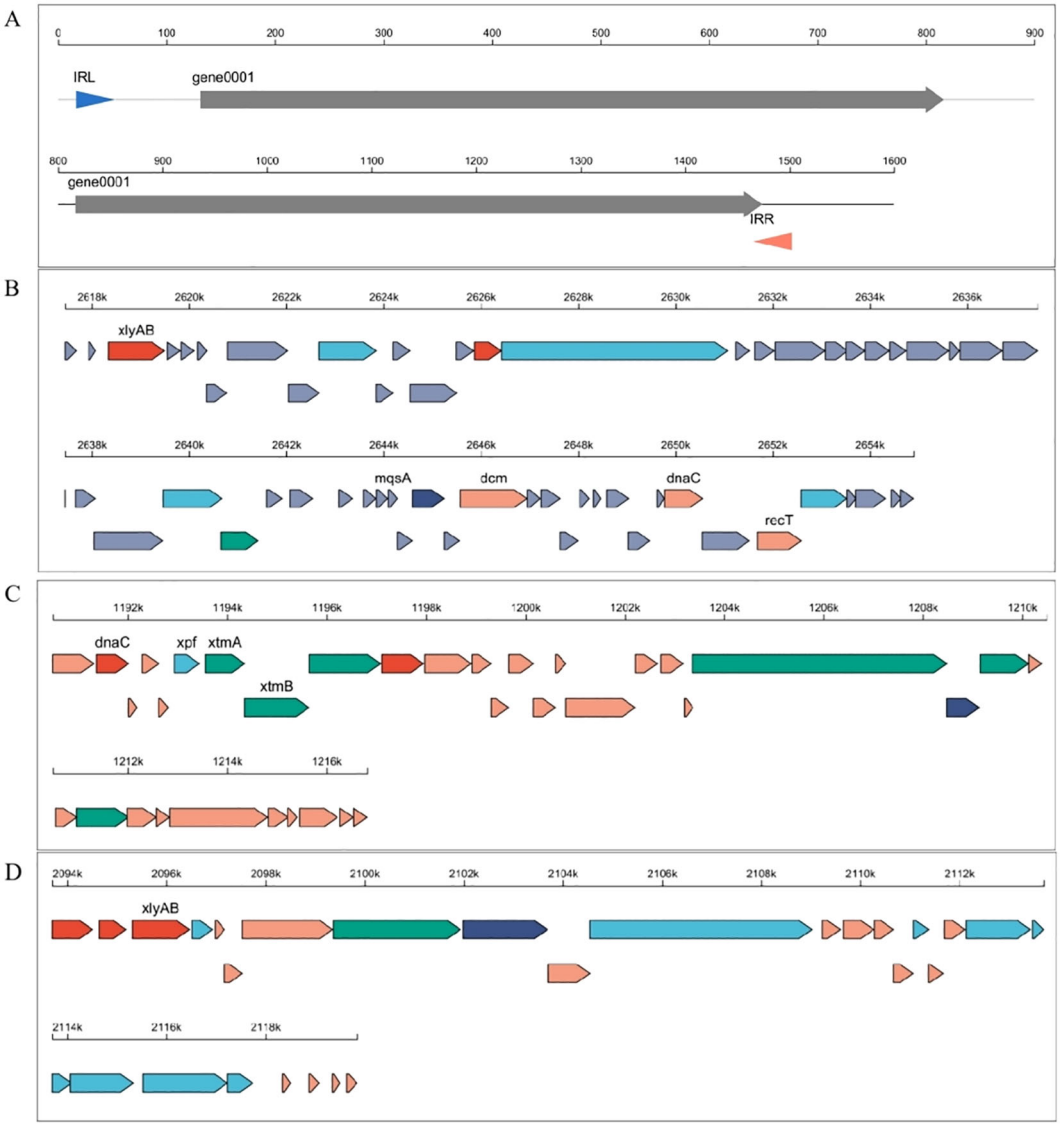
Analysis of mobile genetic elements in the *Bacillus amyloliquefaciens (*XYG6) and *Bacillus aryabhattai (*XAG3) genomes. **(A)** Map of insertion sequence elements in the XAG3 genome; **(B-D)** Linear maps of integrated prophages in the XYG6 genome.

The results of our study show that the whole-genome sequencing of two *Bacillus* strains, *B. aryabhattai* (XAG3) and *B. amyloliquefaciens* (XYG6), which have severe allelopathic effects in the roots of *C. equisetifolia*, revealed genome sizes of 5,507,526 bp and 3,854,088 bp, respectively. The GC content was 38.07% for XAG3 and 46.37% for XYG6. There are seven plasmids indicated in the XAG3 genome, four of which are unknown. The XYG6 genome contains no annotated plasmids. Both the XAG3 and XYG6 genomes had tandem repeated sequences (67 vs. 95, respectively), dispersed repeated sequences (43 vs. 52, respectively), and pseudogenes (206 vs. 170). In addition, one insertion sequence was found in the XAG3 genome, two transposons from the helitronORF and LINE families, and three integrated phage genomes in the XYG6 genome (**Figure 1**; **Supplementary Table 1**). These findings highlight changes in the genome compositions of XYG6 and XAG3 and suggest the possibility of horizontal gene transfer.

**Figure 2:**
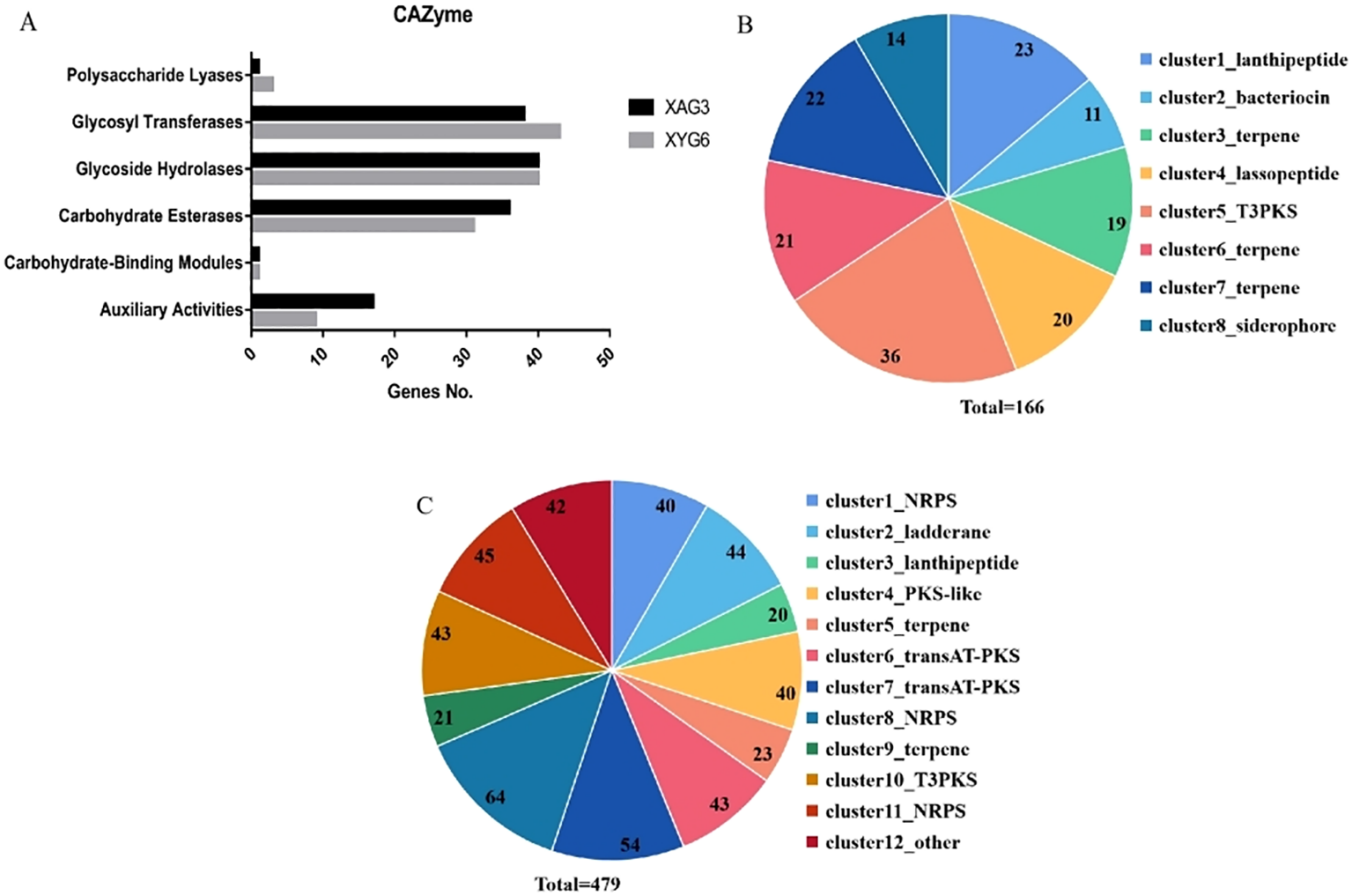
Number of metabolic system-related genes in the XYG6 and XAG3 genomes. Number of genes in each class of carbohydrate-active enzymes in the XAG3 and XYG6 genomes **(A)**; Number of secondary metabolite synthesis gene clusters and corresponding genes in the XAG3 **(B)** and XYG6 **(C)** genomes.

Analysis of metabolic system genes revealed that the XAG3 and XYG6 genomes contain 133 and 127 carbohydrate-active enzyme genes, respectively. **Figure 2A** shows the number of genes in each of the six major categories of carbohydrate-active enzymes. Whereas, secondary metabolite synthesis gene analysis identified 8 and 12 clusters in the XAG3 and XYG6 genomes, including 166 and 479 genes, respectively (**Figures 2B, C**). Similarly, the XAG3 and XYG6 genomes contain gene clusters involved in the synthesis of terpenes, T3PKS, and lanthipeptide (**Supplementary Tables 2, 3**). The difference is in the position of the lanthipeptide synthesis gene cluster; XAG3 is on a plasmid, whereas XYG6 is on the chromosome. This shows that both strains have a large number of secondary metabolite synthesis genes in their genomes, with terpene and T3PKS synthesis genes being especially prominent.

### 2.2 Metabolite analysis of *C. equisetifolia* after *Bacillus* infection

**Figure 3:**
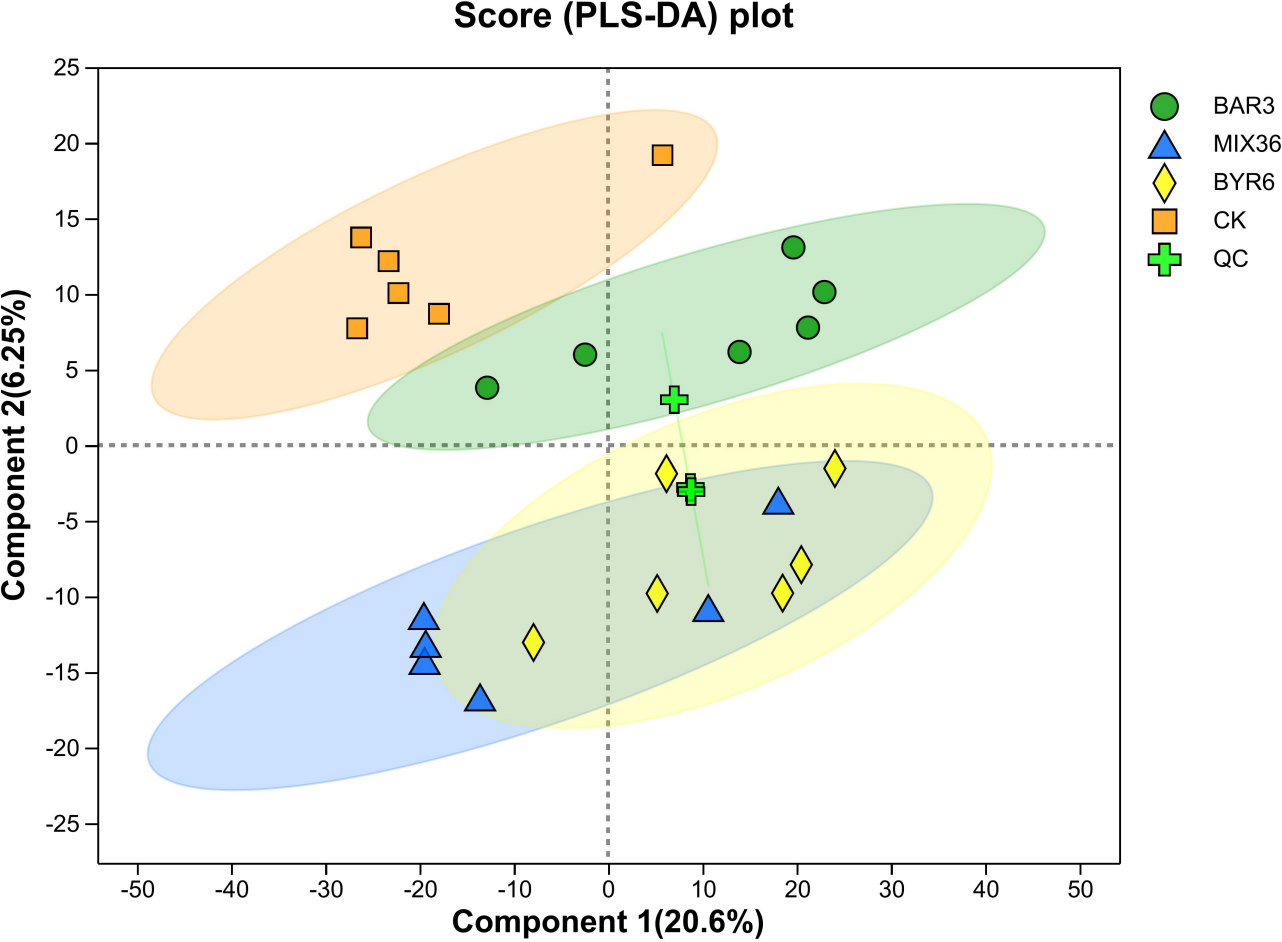
PLS-DA analysis of differences between groups in *C. equisetifolia* samples. Component 1 represents the first principal component and Component 2 represents the second principal component.

To investigate the metabolites, untargeted metabolomics analysis was performed on *C. equisetifolia* seedlings infected with either individual or mixed strains using liquid chromatography-mass spectrometry (LC-MS). Partial least squares discriminant analysis (PLS-DA) revealed that samples from the control (CK) and XAG3-infected (BAR3) groups clustered together. However, samples from the XYG6-infected (BYR6) and XAG3+XYG6-infected (MIX36) groups showed no separation, as shown in **Figure 3**.

**Figure 4:**
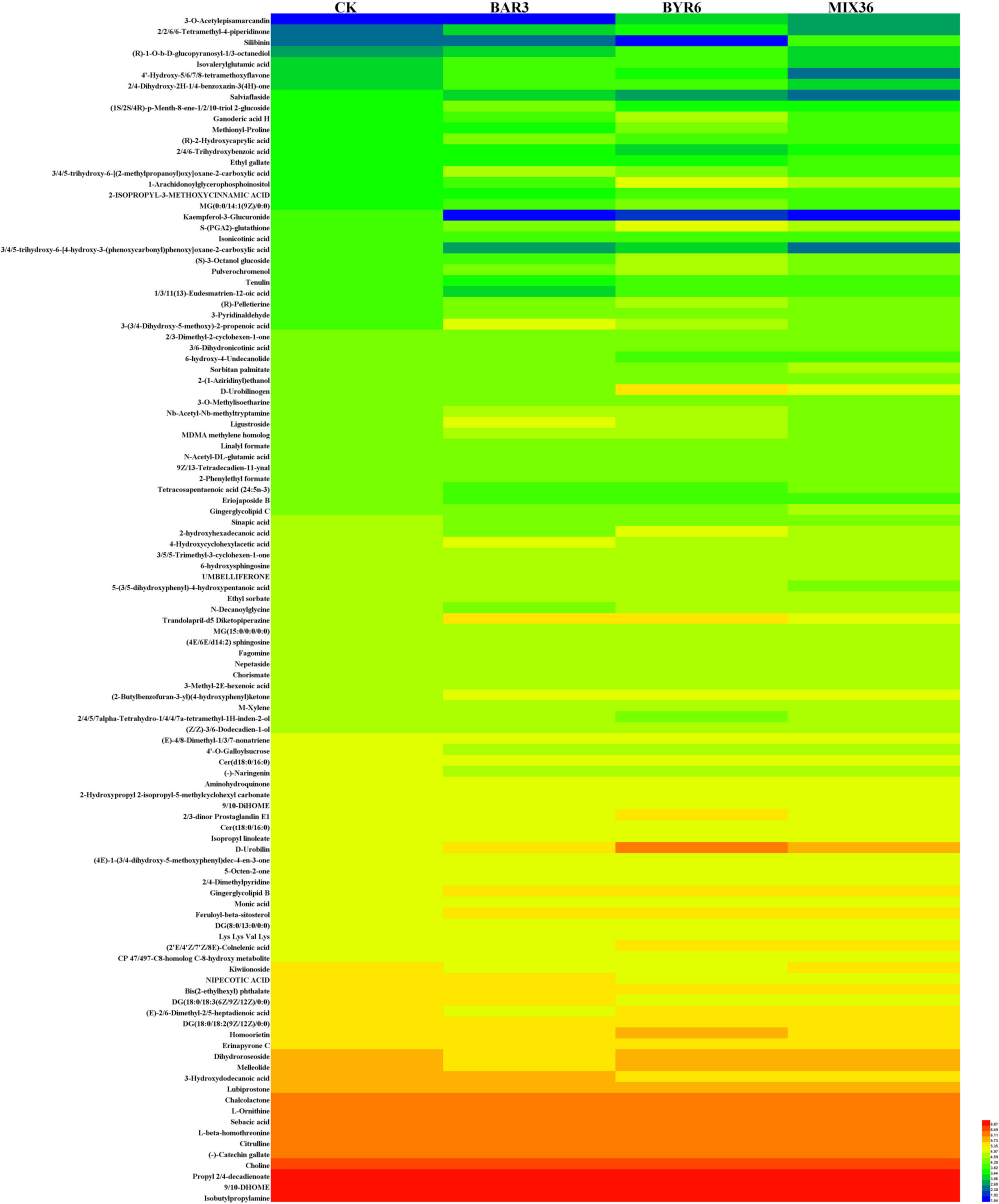
Heat maps of different metabolites of *C. equisetifolia* in different treatment groups.

**Figure 5:**
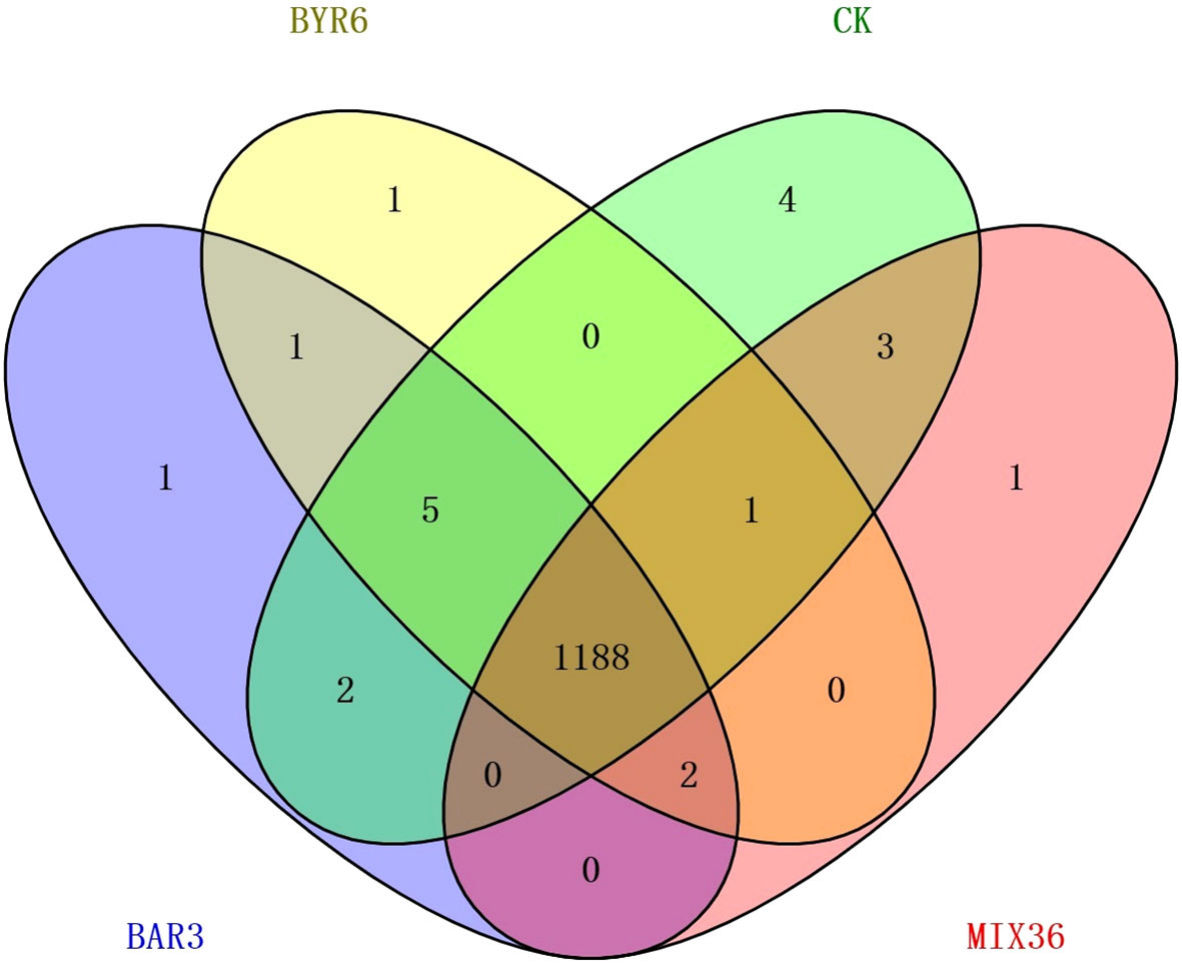
Venn diagram of metabolites common and specific to different treatment groups.

The results of this study demonstrate that 109 metabolites were identified at significantly different levels among the four treatment groups, as shown in **Figure 4** and **Supplementary Table 4**. Compared to the control, 32 and 43 metabolites were significantly up and downregulated in BAR3, 55 and 33 in BYR6, and 35 and 31 in MIX36 treatment groups, respectively. For example, (2-butylbenzofuran-3-yl)(4-hydroxyphenyl)ketone and dihydroxyacetone (dimer) were both significantly upregulated, while (-)-catechin gallate, 12-dehydroporson, 2,4,5,7alpha-tetrahydro-1,4,4,7a-tetramethyl-1H-inden-2-ol, 2-{[hydroxy(5-hydroxy-1H-indol-3-yl)methylidene]amino}acetic acid, austinol, CP 47,497-C8-homolog C-8-hydroxy metabolite, eriojaposide B, isoachifolidiene, and kaempferol-3-glucuronide were all significantly downregulated; 48 and 46 metabolites were significantly upregulated and downregulated in BAR3 and 27 and 13 in BYR6 single-strain infection groups compared to the mixed bacterial infection group. For example, 2,3-dihydro-2,3-dihydroxy-9-phenyl-1H-phenalen-1-one, 3-(3,4-dihydroxy-5-methoxy)-2-propenoic acid, 4’-hydroxy-5,6,7,8-tetramethoxyflavone, 5-(3,5-dihydroxyphenyl)-4-hydroxypentanoic acid, Nb-acetyl-Nb-methyltryptamine, and palmitoyl glucuronide were all significantly upregulated, while ethyl gallate, gingerglycolipid *C, silibinin*, and tanacetol A were all significantly downregulated. Furthermore, we investigated unique metabolites in each group, identifying quercetin-3-glucuronide, kaempferol-3-glucuronide, isoachifolidiene, and 5-aminopentanal for the CK group, prunitrin for the BAR3 group, 5-deoxymyricanone for the BYR6 group, and butyryl-L-carnitine for the MIX36 group (**Figure 5**).

### 2.3 Analysis of gene expression at the transcriptional level in *C. equisetifolia* after *Bacillus* infection

To better understand how *Bacillus* infection influences biosynthesis in *C. equisetifolia*, a transcriptome study was performed on seedlings infected with individual and combined *Bacillus* strains to discover differentially expressed genes/transcripts. The sequencing data collected for the CK, BAR3, BYR6, and MIX36 groups were 39.96 Gb, 39.16 Gb, 37.89 Gb, and 40.11 Gb, respectively. Each sample had sequencing data that exceeded 6 Gb, and the error rate was less than 0.0266%. In each sample, the fraction of bases with a sequencing quality of more than 99% exceeded 97.47%, with those with quality greater than 99.9% exceeding 92.83%. The CK, BAR3, BYR6, and MIX36 groups had an average GC content of 47.28%, 47.3%, 46.99%, and 46.98%, respectively. The GC concentration of each sample ranged between 46.67% and 47.46%, as indicated in **Supplementary Table 5**. This shows that the transcriptome sequencing data volume, error rate, base quality, and GC content are adequate and dependable.

**Figure 6:**
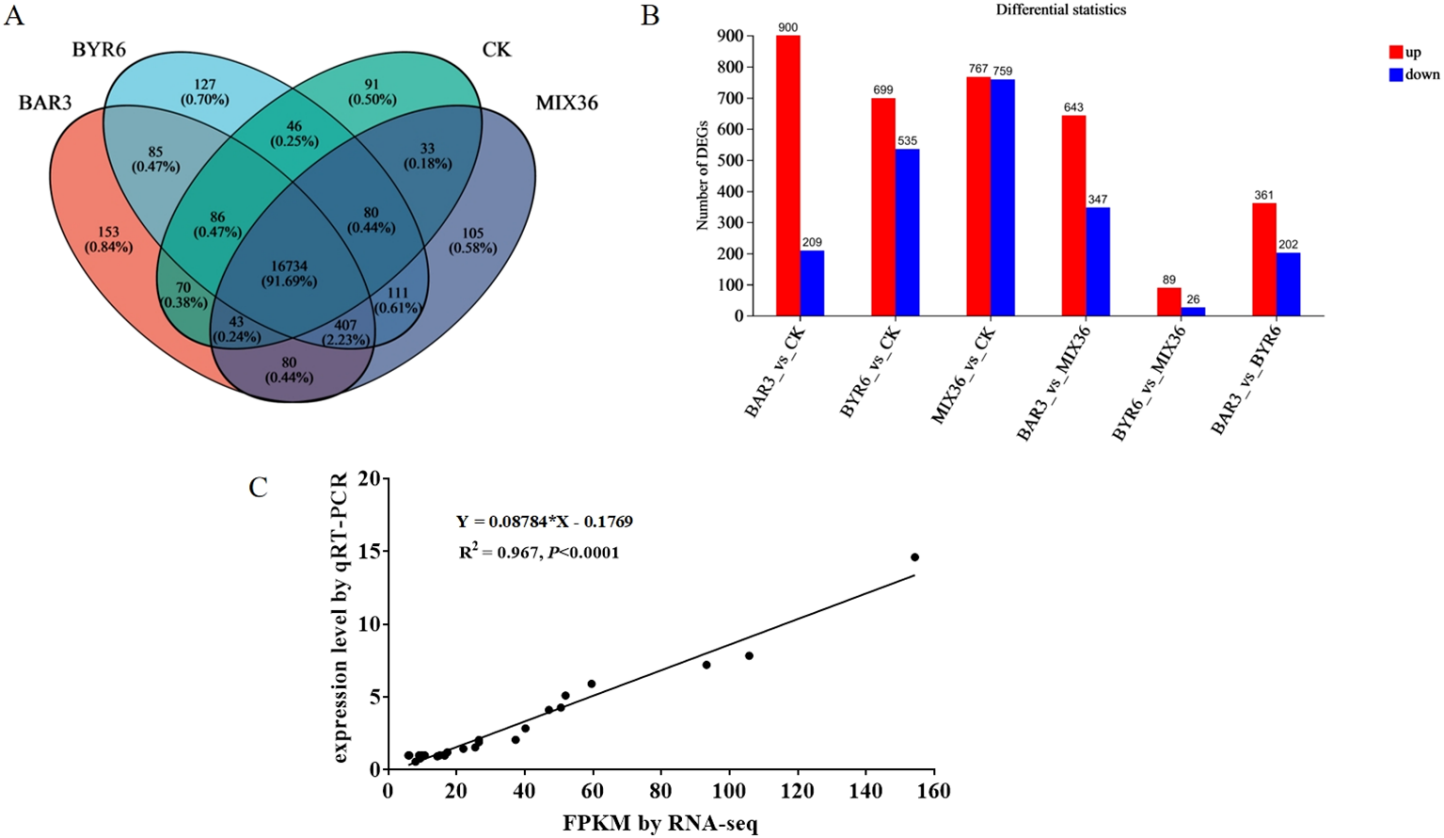
Differentially expressed genes in *C. equisetifolia* after *Bacillus* infection. **(A)** Venn diagram of differentially expressed genes/transcripts; **(B)** Numbers of significantly upregulated and downregulated genes/transcripts among the different groups; **(C)** Correlation analysis between transcriptomic sequencing data and qRT-PCR data.

Further analysis revealed a total of 16,734 common genes/transcripts in the CK, BAR3, BYR6, and MIX36 groups, with 91, 153, 127, and 105 unique genes/transcripts, respectively (**Figure 6A**). In comparison to the control, the BAR3, BYR6, and MIX36 groups had 1,109, 1,234, and 1,526 differentially expressed genes/transcripts, respectively. Among them, 900, 699, and 767 genes/transcripts were elevated, whereas 209, 535, and 759 were downregulated. *Bacillus* infection resulted in significant variations in gene expression in *C. equisetifolia* seedlings. Furthermore, gene expression in *C. equisetifolia* seedlings infected with individual and mixed strains varied significantly. **Figure 6B** shows that the numbers of differentially expressed genes/transcripts in the BAR3 and BYR6 groups were 990 and 115, respectively, with 643 and 89 upregulated and 347 and 26 downregulated. The BYR6 and MIX36 groups differed just slightly in terms of upregulated and downregulated genes.

In addition, the transcriptome sequencing results were confirmed by randomly picking differentially expressed genes from the transcriptomic analysis for validation using qRT-PCR. Gene expression levels determined by qRT-PCR were consistent with transcriptome sequencing results. **Figure 6C** displays a correlation study (R^^2 =^ 0.967, *P*<0.0001) that confirms the reproducibility and repeatability of the transcriptome sequencing data.

**Figure 7:**
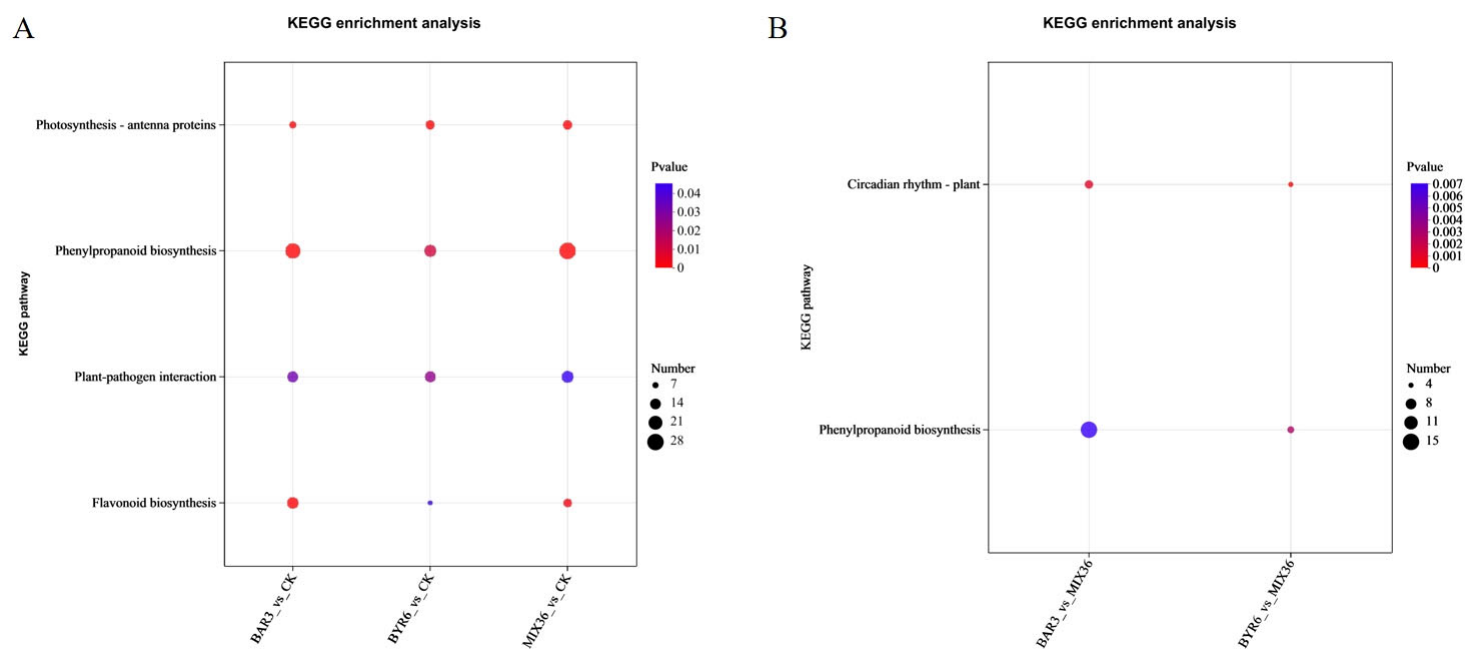
KEGG functional enrichment analysis of differentially expressed genes/transcripts in *C. equisetifolia* after *Bacillus* infection. The bubble chart displays the top 20 enriched pathways; KEGG enrichment analysis of differentially expressed genes/transcripts **(A)** in BAR3_vs_CK, BYR6_vs_CK, and MIX36_vs_CK and **(B)** in BAR3_vs_MIX36 and BYR6_vs_MIX36.

**Figure 8:**
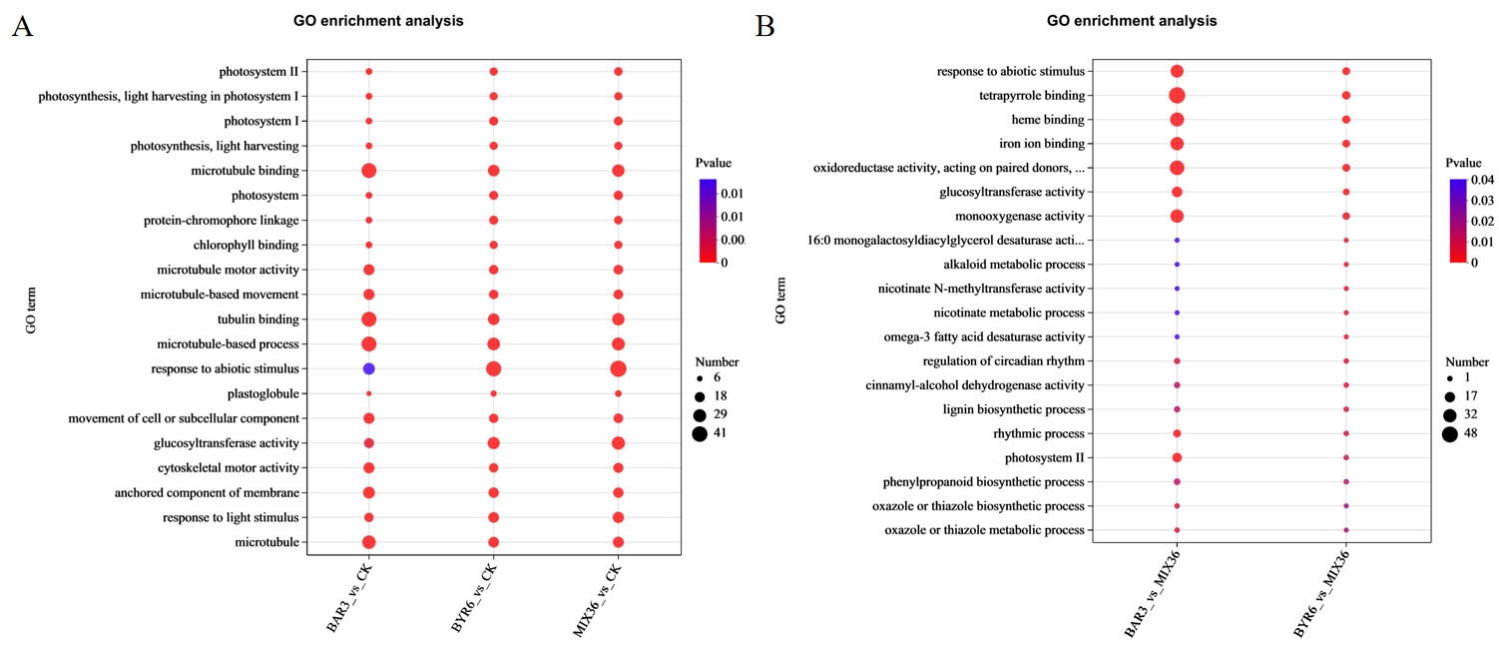
GO functional enrichment analysis of differentially expressed genes/transcripts in *C. equisetifolia* after *Bacillus* infection. The bubble chart displays the top 20 enriched pathways; GO enrichment analysis of differentially expressed genes/transcripts **(A)** in BAR3_vs_CK, BYR6_vs_CK, and MIX36_vs_CK and **(B)** BAR3_vs_MIX36 and BYR6_vs_MIX36.

Further functional enrichment analysis of the Kyoto Encyclopedia of Genes and Genomes (KEGG) revealed that differentially expressed genes/transcripts following *Bacillus* infection were primarily enriched in pathways such as photosynthesis - antenna proteins, flavonoid biosynthesis, phenylpropanoid biosynthesis, and plant-pathogen interaction (**Figure 7A**). The differentially expressed genes/transcripts between single- and mixed-strain infections mostly occurred in pathways such as circadian rhythm - plant and phenylpropanoid biosynthesis (**Figure 7B**). GO analysis revealed that differentially expressed genes/transcripts following *Bacillus* infection were primarily enriched in pathways such as photosystem, photosynthesis, light harvesting, microtubule binding, and microtubule-based movement, and microtubule (**Figure 8A**). Similarly, the differentially expressed genes/transcripts between single- and mixed-strain infections were primarily enriched in pathways such as tetrapyrrole binding, response to abiotic stimuli, iron ion binding, and heme binding (**Figure 8B**). Differentially expressed genes/transcripts after *Bacillus* infection were significantly enriched in metabolic pathways for secondary metabolite biosynthesis and breakdown, implying that *Bacillus* may influence *C. equisetifolia* secondary metabolite biosynthesis and catabolism by regulating gene expression at the transcriptional level.

### 2.4 Differentially expressed genes in secondary metabolite biosynthetic pathways

**Figure 9:**
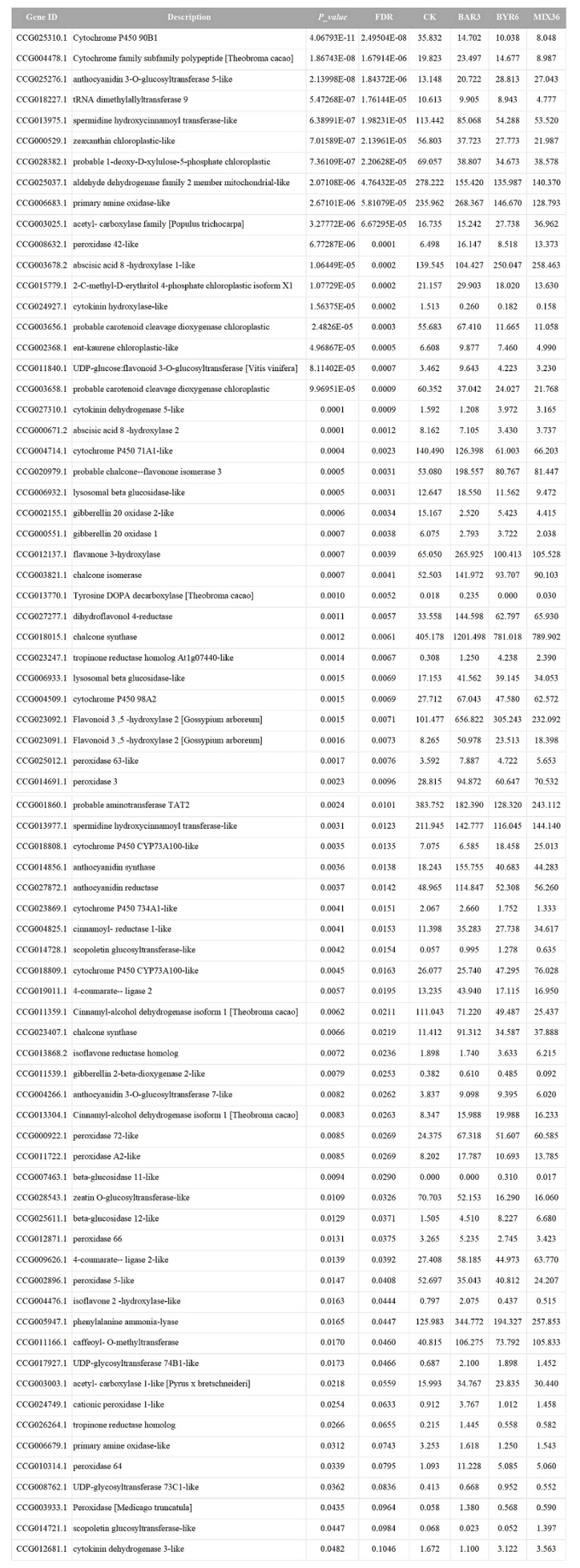
Potential genes that *Bacillus* might assist the host *C. equisetifolia* in regulating.

**Figure 10:**
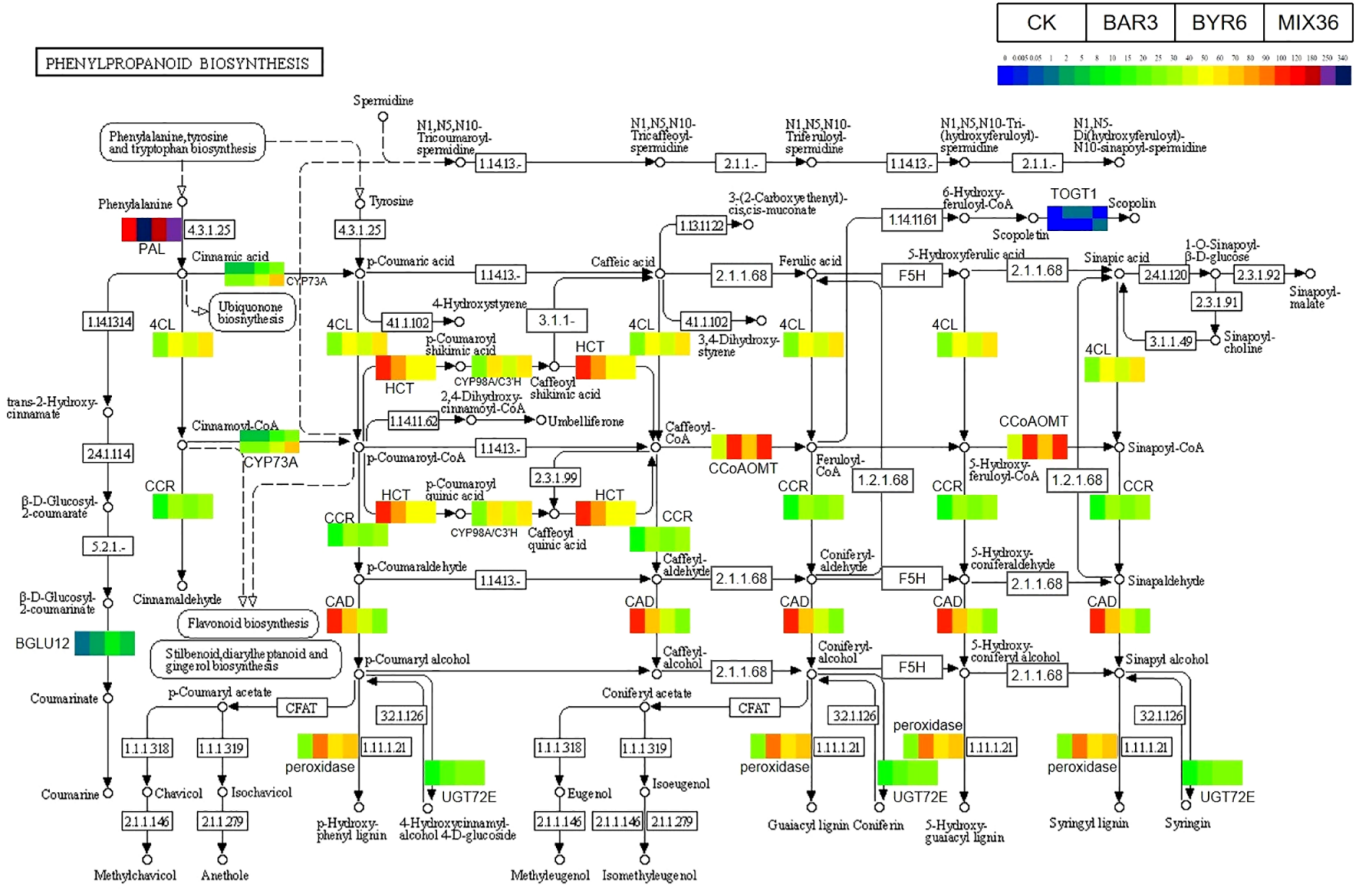
Expression analysis of *C. equisetifolia* genes related to the Phenylpropanoid biosynthesis pathway following *Bacillus* infection. The KEGG path map is derived from Kanehisa Laboratories (Kanehisa and Goto, 2000; Kanehisa, 2019; Kanehisa et al., 2023).

**Figure 11:**
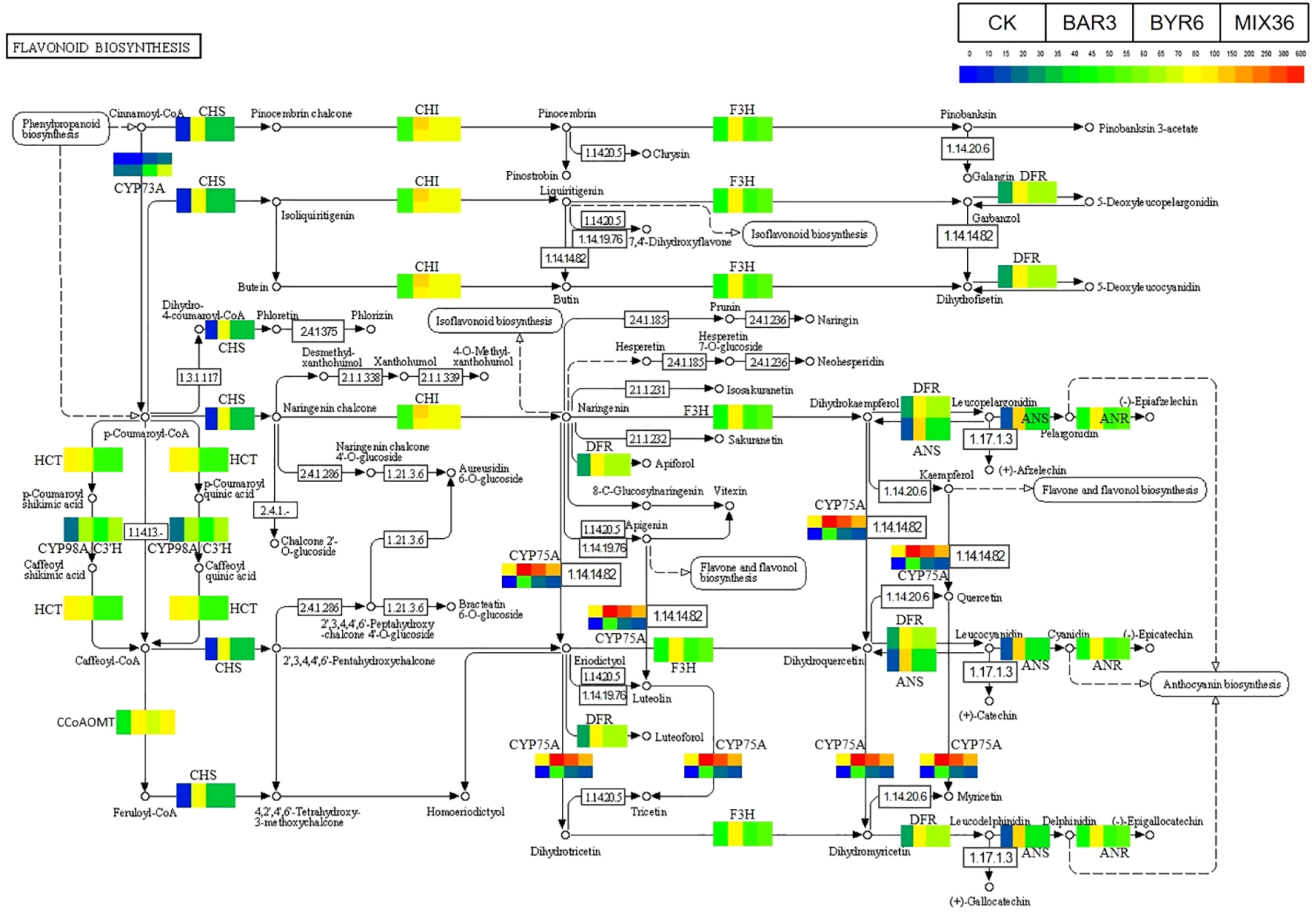
Expression analysis of *C. equisetifolia* genes related to the Flavonoid biosynthesis pathway following *Bacillus* infection. The KEGG path map is derived from Kanehisa Laboratories (Kanehisa and Goto, 2000; Kanehisa, 2019; Kanehisa et al., 2023).

Further results show that DESeq2 differential expression analysis and KEGG annotation analysis revealed that 74 differentially expressed genes/transcripts regulate secondary metabolite biosynthesis pathways (**Figure 9**). Metabolomics and transcriptomic analysis of *C. equisetifolia* showed the 11 important secondary metabolite biosynthesis among them alkaloids biosynthesis, phenylpropanoid biosynthesis, phenylpropanoid, terpenes biosynthesis and related genes were putatively regulated. Following *Bacillus* infection, the differentially expressed genes in *C. equisetifolia* were primarily enriched in the phenylpropanoid and flavonoid biosynthesis pathways, with genes such as BGLU12, CCR, TOGT1, CYP75A, and CHS being considerably elevated (**Figures 10**, **11**). Similarly, gene expression in *C. equisetifolia* in these two secondary metabolite biosynthetic pathways was similar after infection by *B. amyloliquefaciens* and the mixed strains; however, gene expression in *C. equisetifolia* infected by *B. aryabhattai* and the mixed strains differed significantly, with genes such as ANR, ANS, CAD, CHI, CYP75A, DFR, and F3H significantly upregulated and CYP73A, CYP93B2_16, and TOGT1 significantly downregulated.

### 2.5 Correlation analysis of secondary metabolite synthesis-related genes and differential metabolites

**Figure 12:**
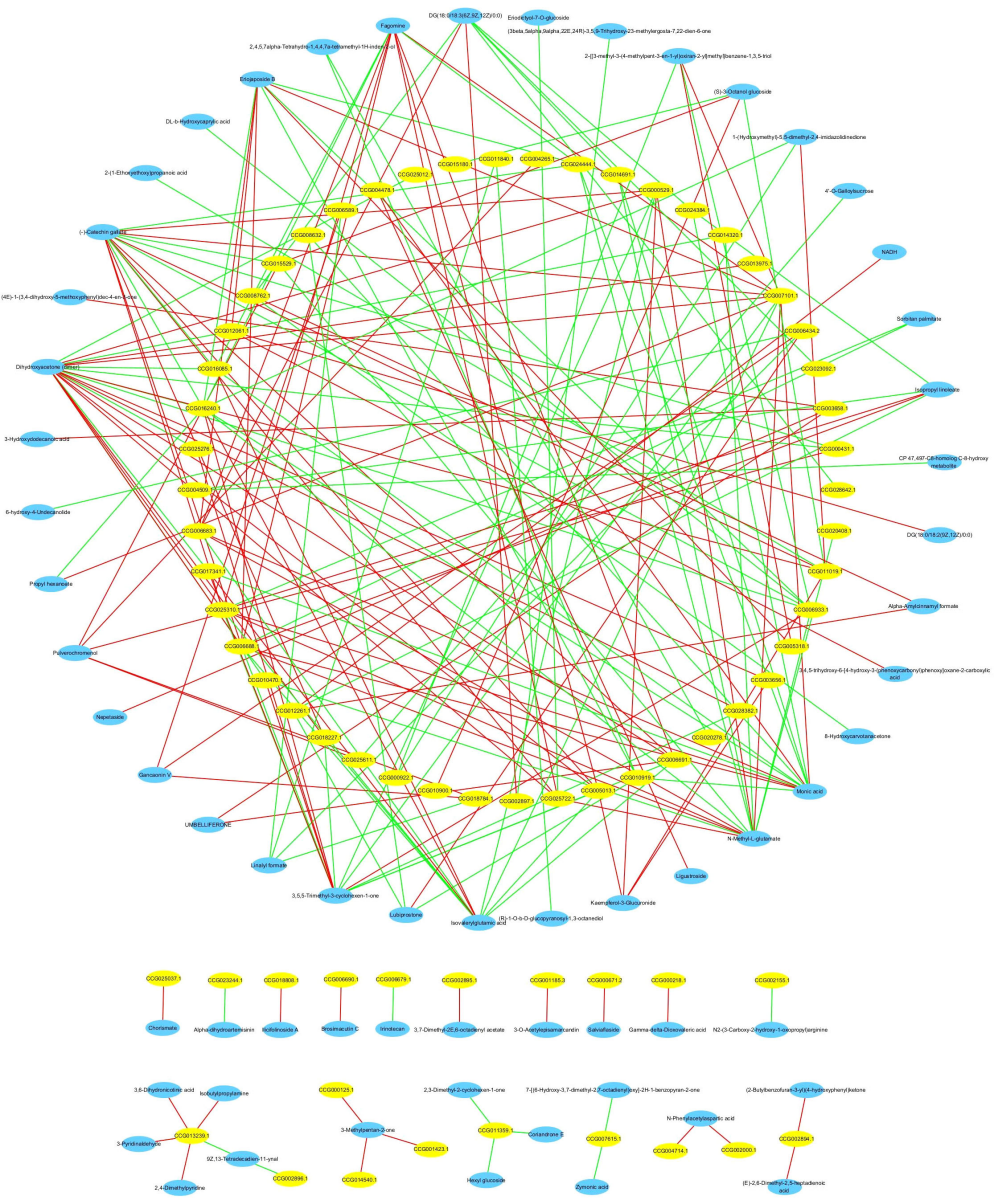
Network diagram of the correlation analysis between differential metabolites and differentially expressed genes of *C. equisetifolia* after *Bacillus* infection. All absolute correlation coefficients ≥0.9, and all *P*-values <0.01.

We performed a correlation analysis of genes and metabolites that differed after *Bacillus* infection to identify the mechanism underlying the transcriptional control of differential metabolites from the secondary metabolite biosynthesis and metabolic pathways. The association analysis revealed that 63 differential metabolites had a high link with 69 genes/transcripts involved in secondary metabolite biosynthesis pathways. 42 and 36 differential metabolites had significant positive and negative associations with 48 differentially expressed genes (absolute correlation coefficient ≥0.9 and *P*<0.01; **Figure 12**). Interestingly, (-)-catechin gallate had a high positive association with CCG007101.1 (Pearson correlation coefficient [PCC]=0.972, *P*<0.01) and a strong negative correlation with CCG016240.1 (PCC=-0.952, *P*<0.01). Dihydroxyacetone (dimer) had a high positive connection with CCG000529.1 (PCC=0.956, *P*<0.01) and CCG007101.1 (PCC=0.95, *P*<0.01), but a strong negative correlation with CCG006933.1 (PCC=-0.954, *P*<0.01) and CCG016240.1 (PCC=-0.966, *P*<0.01).

**Figure 13:**
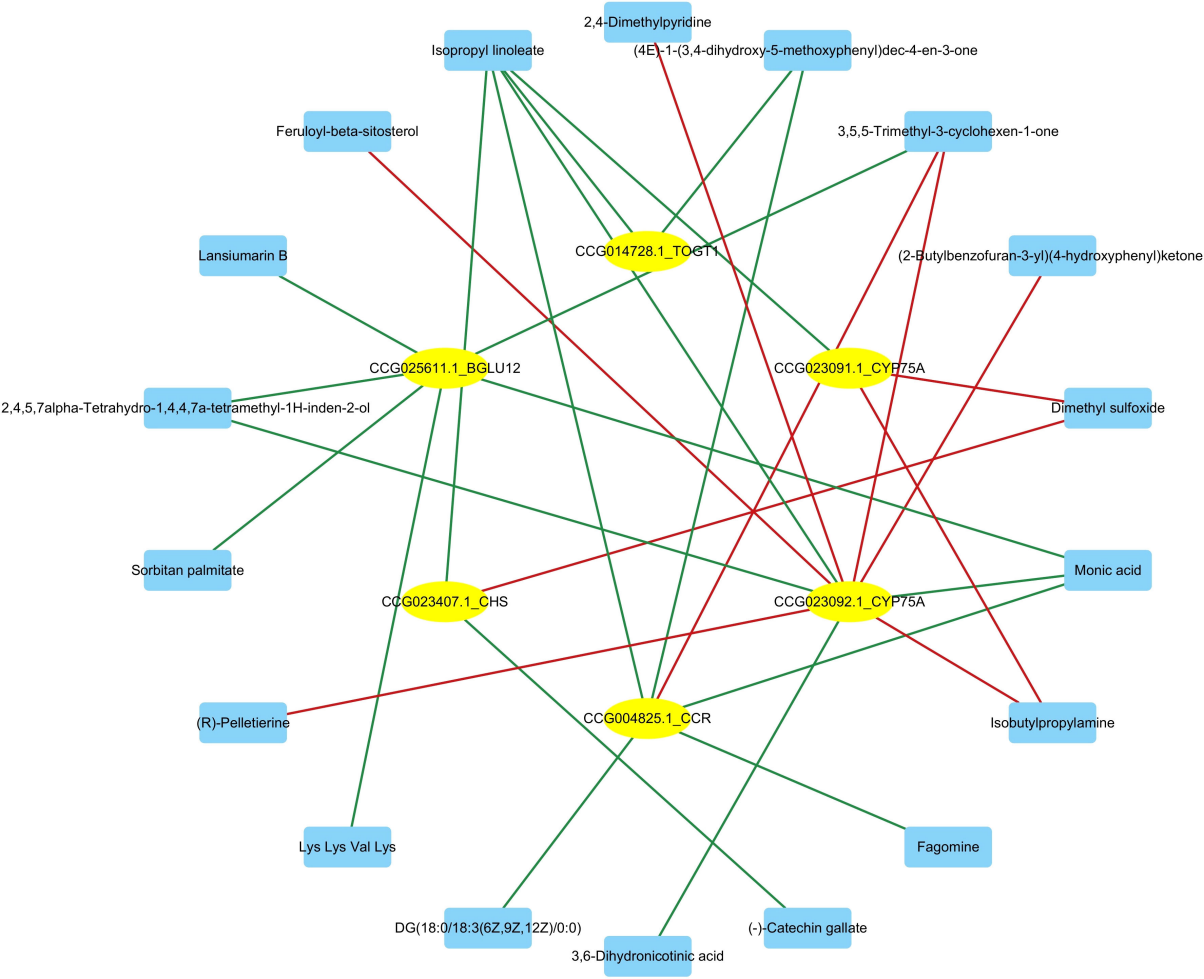
Correlation analysis between differential metabolites and the genes significantly upregulated (*BGLU12, CCR, TOGT1, CYP75A*, and *CHS*) of *C. equisetifolia* after *Bacillus* infection.

To further comprehend the regulatory genes involved in secondary metabolite biosynthesis, a correlation analysis was conducted between the differential metabolites and the genes significantly enriched in secondary metabolite biosynthesis pathways following *Bacillus* infection in *C. equisetifolia*, including BGLU12, CCR, TOGT1, CYP75A, and CHS. **Figure 13** the results of correlation analysis show that a total of 18 metabolites were associated with these genes.

## 3 Discussion

Plants and microorganisms coexist in the ecosystem, and their communication on the earth forms a complicated network. Endophytic bacteria in plants contribute to maintaining normal physicochemical characteristics. Under stress, these bacteria can directly or indirectly promote plant growth and development by producing hormones, enzymes, phytochemicals, and iron transporters as well (Khan and Ali, 2022). In this study, genomic sequencing results for *B. amyloliquefaciens* and *B. aryabhattai* revealed the presence of insertion sequences or transposons in both strains. In a previous study, it was reported that these types of strains may use horizontal gene transfer to assist bacteria overcome obstacles or achieve ecological advantages (Brito, 2021; Arnold et al., 2022). Additionally, it could also assist in safeguarding the host against other biological invasions and facilitate adaptation to new environments (Husnik and McCutcheon, 2018).

To assess the impact of endophytic Bacillus strains—*Bacillus aryabhattai* (BAR3 group) and *Bacillus amyloliquefaciens* (BYR6 group)—on the metabolites of *C. equisetifolia*, we performed a non-targeted metabolomics analysis, which revealed 109 differential metabolites. Present results indicated the *Bacillus* BAR3 group predominantly affects the production of metabolites such as dihydroxyacetone, austinol, eriojaposide B, kaempferol-3-glucuronide, and (-)-catechin gallate in the host *C. equisetifolia*. Following *Bacillus* colonization, the expression of dihydroxyacetone was significantly elevated. Similar to our findings, prior research has demonstrated that dihydroxyacetone, once phosphorylated, creates dihydroxyacetone phosphate (DHAP), which enters the metabolic pathway and influences processes such as metabolic efficiency and DNA repair (Mehta et al., 2021). It was also shown to increase the respiration rate of microbial communities in saline solutions (Oren, 2016). Similarly, the further study indicated that following *Bacillus* colonization, the levels of certain metabolites in *C. equisetifolia*, such as austinol, eriojaposide B, kaempferol-3-glucuronide, and (-)-catechin gallate, significantly decreased Austinol (Matsuda et al., 2013) and eriojaposide B (Ito et al., 2001) are known to be terpenoid compounds. In addition, kaempferol-3-glucuronide belongs to flavonols, and its reduction may be attributed to the stronger competitive advantage of kaempferol-3-glucoside to kaempferol-3-glucuronide, which directs more substrates toward glycosides (Yao et al., 2021). Kaempferol-3-glucoside can also synthesize the allelochemical kaempferol-3-O-D-glucoside (Li et al., 2011). (-)-Catechin gallate can inhibit membrane-localized K^+^ channels and restrict Ca^2+^ entry into the guard cells of *Arabidopsis*, inhibiting abscisic acid (ABA)-induced stomatal closure and increasing surface temperatures (Mushtaq et al., 2021; Sato et al., 2022). As tea gardens age, various allelochemicals such as epicatechin, catechin, and epicatechin gallate accumulate in the tea rhizosphere. Elevated concentrations of these allelochemicals, including various active catechins, significantly inhibit *Bacillus* growth, promoting plant growth (Mushtaq et al., 2019; Arafat et al., 2020). Considering the decrease in (-)-catechin gallate level in *C. equisetifolia* observed after *Bacillus* infection in this study, it is clear that there are interactions between growth-promoting bacteria and allelochemicals.

We conducted transcriptome sequencing analysis to determine the genes related to secondary metabolite biosynthesis that *Bacillus aryabhattai* and *Bacillus amyloliquefaciens* might aid the host *C. equisetifolia* regulate; we identified 74 key candidate genes involved in secondary metabolite biosynthesis. BGLU12, CCR, TOGT1, CYP75A, and CHS of *C. equisetifolia* were highly expressed following *Bacillus aryabhattai* and *Bacillus amyloliquefaciens* colonization. Similar to our findings, a previous study reported that BGLU12-like proteins alter the cell wall by modifying hemicellulose and cellulose (Yu et al., 2019). In contrast to our findings, another study reported that after introducing compound bacteria to wheat, the expression of the CCR and TOGT1 genes in leaves was downregulated (Ji et al., 2023). This discrepancy might be attributed to differences in tissue types and the specific bacterial species introduced. For example, gene expression in roots and leaves may be different. In our study, the expression of CCR in *C. equisetifolia* treated with mixed bacteria was lower than that with BAR3 single strain treatment yet higher than that with BYR6 single strain treatment. Flavonoid 3’5’-hydroxylase (CYP75A) plays a role in the biosynthesis of most secondary metabolites in plants to counter biotic and abiotic stresses (Xiao et al., 2021). Chalcone synthase (CHS) has a wide range of substrates, and its enzymatic activity and expression levels can significantly influence the biosynthesis of flavonoid compounds (Tong et al., 2021). The absence of the flavonoid biosynthesis genes CHS, CHI, and CHIL can alter rice’s flavonoid and lignin profiles (Lam and Wang, 2022). CHS expression is related to allelochemicals derived from *Bacillus* (Guo et al., 2011). By upregulating these genes, endophytes promote the host plant’s resilience to infectious agents and pests, helping to protect the forest. Additionally, CHS and dihydroflavonol reductase (DFR) are genes involved in flavonoid synthesis and heavy metal resistance in the leaves of *Broussonetia papyrifera*. The overexpression of CHS and DFR promotes the accumulation of flavonoids (Peng, 2022). These findings suggest that *Bacillus* can support the host *C. equisetifolia* by regulating the expression of genes in the secondary metabolite biosynthesis pathways. *Bacillus amyloliquefaciens* and *Bacillus aryabhattai* improve the synthesis of these compounds by modifying crucial enzymatic pathways in the host plant, allowing *Casuarina equisetifolia* a competitive advantage while promoting a more sustainable forest ecosystem.

The intercorrelation between the biosynthesis genes of secondary metabolites and the metabolism of *C. equisetifolia* was elucidated using correlation analysis. Among these, 69 differentially expressed genes are related to synthesizing 63 metabolites in *C. equisetifolia*. Specifically, CMBL, ZEP/ABA1, VTE3/APG1, CYP450 86B1-like, CYP76B10/G10H, miaA/TRIT1, CYP90B1/DWF4, and HPL are positively correlated with the allelochemical (-)-catechin gallate, whereas SKU5, bglX, AAE3, LAC6, and Bp10 correlate negatively. CMBL might be involved in the shortened pathway for phenol biodegradation, potentially aiding in establishing a new phenol catabolic pathway through the dienelactone hydrolase (He et al., 2022). Prior investigations by our group revealed that soil, litter, and roots of *C. equisetifolia* contained allelochemicals such as 2,4-di-tert-butylphenol, and the majority of compounds found in methanol extracts of *C. equisetifolia* roots from different aged forests were phenols (Yao et al., 2015). In our earlier investigation, we identified the major components in extracts from *C. equisetifolia* roots/litters of varying ages, as well as the fermentation broth of allelopathic endophytic bacteria *B. amyloliquefaciens* and *B. aryabhattai*. Similar to the metabolites discovered in this investigation, all included phenolic acids. For example, sinapyl alcohol has previously been identified in the fermentation broth of endophytic *Bacillus* strains from *C. equisetifolia* (Zhang et al., 2020). In the current study, we observed that the concentration of allelochemical sinapic acid decreased after infection of *C. equisetifolia* by endophytic *B. amyloliquefaciens* and *B. aryabhattai* strains and had a negative relationship with peroxidase genes/transcripts. Previously, our group extracted kaempferol from *C.equisetifolia* litter (Haisheng et al., 2018). Kaempferol can be changed to kaempferol-3-glucuronide. In the present study, after infecting *C. equisetifolia* with endophytic *Bacillus*, *B. amyloliquefaciens* and *B. aryabhattai* strains, the level of kaempferol-3-glucuronide decreased and showed positive relationships with ZEP/ABA1, NCED, VTE3/APG1, and CYP450 86B1-like genes/transcripts. Ornithine was earlier discovered in the fermentation broth of endophytic *B. amyloliquefaciens* and *B. aryabhattai* strains from *C. equisetifolia*. Ornithine can be converted into pyrrolizidine, an allelochemical alkaloid. In this study, after infecting *C. equisetifolia* with endophytic *Bacillus* strains, the L-ornithine level decreased and was inversely linked with the expression of the CYP736A gene. This suggests that *Bacillus* might regulate the release of allelochemicals by assisting their host, *C. equisetifolia*, in regulating biosynthesis and catabolism-related genes.

## 4 Conclusions

In conclusion, the investigation of endophyte-mediated allelopathy in *Casuarina equisetifolia* indicates a complicated and beneficial interaction between the host plant and its microbial partners, specifically *Bacillus amyloliquefaciens* and *Bacillus aryabhattai*. These endophytes have a major influence on the host’s gene expression, activating defense-related pathways and increasing the generation of secondary compounds with allelopathic qualities. By controlling these pathways, endophytes assist the plant in suppressing competitive plants and protecting against diseases, enhancing forest health and resilience. The findings show potential for using endophytic bacteria to naturally promote allelopathic traits in plants, providing a more sustainable approach to forest management and conservation. Additional research into these symbiotic connections may provide new techniques for strengthening plant defense mechanisms and environmental stability. Thus, endophytes provide an effective strategy for increasing the ecological competitiveness and sustainability of forest species such as *Casuarina equisetifolia*.

## 5 Materials and methods

### 5.1 Isolation and whole genome sequencing of *Bacillus amyloliquefaciens* and *Bacillus aryabhattai*

Endophytic *B. amyloliquefaciens* and *B. aryabhattai* were previously isolated and purified from the roots of *C. equisetifolia* by our research group. These strains were identified based on colony morphology, Gram staining, and other strain identification methods (Huang, 2019). The strains *B. amyloliquefaciens* and *B. aryabhattai* were designated XYG6 and XAG3, respectively, and subsequently preserved. Genomic DNA from XYG6 and XAG3 was extracted using the CTAB method, and whole genome sequencing was performed through a combination of Illumina Hiseq and PacBio single-molecule sequencing platforms. Sequencing data were analyzed using various software and databases, including Phage_Finder, ISEScan, TransposonPSI, and the Carbohydrate Active Enzyme database.

### 5.2 Infection and colonization of *Bacillus amyloliquefaciens* and *Bacillus aryabhattai* and their interaction with aseptic *C. equisetifolia* seedlings

A total of 100 g of *C. equisetifolia* forest soil was sterilized under high pressure (121°C, 60 min) and subsequently allocated to sterile culture bottles. Mature *C. equisetifolia* seeds were chosen, washed with a 1% NaClO solution for 3 min, thoroughly rinsed five times with sterile water, placed in an electric blast drying oven at 60°C for 15 min, and then relocated to the culture bottles filled with sterile soil. These seeds were incubated in an artificial climate incubator with a 12 h/12 h light cycle. Once the seedlings reached approximately 10 cm in height, uniformly grown sterile *C. equisetifolia* seedlings were chosen and randomly divided into four groups, each containing 100 seedlings: (1) CK group, with roots drenched with sterile water; (2) BAR3 group, with roots drenched with a *Bacillus aryabhattai* XAG3 suspension; (3) BYR6 group, with roots drenched with a *Bacillus amyloliquefaciens* XYG6 suspension; (4) MIX36 group, with roots drenched with a mixed suspension of XAG3 and XYG6 at a concentration of 1×10^6^ CFU/mL. Three days’ post root drench treatment, *C. equisetifolia* seedlings were harvested for transcriptome, metabolome, and quantitative PCR assessments, with six replicates for each group.

### 5.3 Transcriptome sequencing analysis of *C. equisetifolia* aseptic seedlings and *Bacillus*-interacting seedlings

Total RNA was extracted from the tissues of *C. equisetifolia* seedlings. The RNA’s integrity, purity, and concentration were assessed using agarose gel electrophoresis and Nanodrop2000. High-quality total RNA samples were chosen for library preparation and quantification, and sequencing was performed on the Illumina platform. We use software such as Fastx_toolkit (Version 0.0.14) and Fastp (Version 0.19.5) for sequencing data quality control. The reference genome is Casuarina_equisetifolia (Version fafu_v1), and the source of the reference genome is http://forestry.fafu.edu.cn/db/Casuarinaceae/index.php. Sequence alignment was performed using Bowtie2 (Version 2.4.1), Hisat2 (Version 2.1.0), TopHat (Version v2.1.1), and STAR (Version 2.7.1a) software, and sequence extraction was performed using Bedtools (Version 2.27.1) software. Use Stringtie (Version 2.1.2) and Cufflinks (Version 2.2.1) software for transcriptome assembly. Gene annotation was performed using Pfam (Version 34.0), KEGG (Version 2021.09), EggNOG (Version 2020.06), Swiss pro (Version 2021.06), NCBI (Version 2021.09), GO (Version 2021.0918), NR (Version 2021.10), and PIR idmapping (Version 2021.06) databases. After obtaining the Read Counts of genes/transcripts, differentially expressed genes/transcripts between samples or between groups were analyzed using DESeq2 (Version 1.24.0), DEGseq (Version 1.38.0), or EdgER (Version 3.24.3) software to identify the differentially expressed genes/transcripts (Robinson et al., 2010; Wang et al., 2010).

### 5.4 Metabolome analysis of aseptic *C. equisetifolia* seedlings and *Bacillus*-interacting seedlings

Twenty-four *C. equisetifolia* seedling samples weighing 50 mg were obtained and sourced from the same region. These samples were transferred to a 1.5 mL EP tubes and 0.5 mL of methanol aqueous solution with a volume ratio of 4:1 containing 0.02 mg/mL L-2-chlorophenylalanine internal standard was added. The grinding machine parameters are set to negative 20 °C, 50 Hz, and 3 min. The sample was put into a grinder for grinding, 200 µL chloroform was added, ultrasonic treatment was performed for 30 min, and the sample was left for 30 min at negative 20°C. Centrifuge at 4°C for 15 min at 13000 g rotation speed. The supernatant was taken and put into a glass derived bottle for drying with nitrogen, then 80 µL of methylxylamine hydrochloride pyridine solution of 15 mg/mL was added, swirled for 2 min, and oscillated for 90 min at 37°C. After oximation, 80 µL of BSTFA derived solution containing 1% TMCS was added, swirled for 2 min, heated at 70°C for 60 min, and then left for 30 min at room temperature. Plant samples were identified by ultra-high resolution mass spectrometer (Q-Exactive, Thermo) and ultra-high performance liquid chromatography (Vanquish H, Thermo).

KEGG and HMDB databases were used to compare metabolites and obtain the annotation information of metabolites in the database. Univariate statistical analysis (t test) combined with multivariate statistical analysis (OPLS-DA/PLS-DA) and multiple change value (FC) were used to screen differential metabolites. The default screening conditions were *P*<0.05 and VIP>1 and (FC<1 or FC>1, FC was not screened by default). The KEGG pathway enrichment analysis defaults to using the BH method to correct for P-values. When the corrected *P*-value is less than 0.05, it is considered that there is significant enrichment in this pathway.

### 5.5 Transcriptomic and metabolomic correlation analysis of aseptic *C. equisetifolia* seedlings and *Bacillus*-interacting seedlings

Transcriptomic and metabolomic data were matched correspondingly for each sample. Differentially expressed genes were determined using the DESeq2 software with the Benjamini-Hochberg (BH) procedure for multiple testing corrections. The criteria for identifying differentially expressed genes were: padjust < 0.05 and fold change > 2. Six differentially expressed gene sets were obtained from the analysis. Differential metabolites were identified using the two-tailed Student’s t-test (unpaired) with the criteria: p-value < 0.05, VIP_pre_PLS-DA > 1, and fold change >1, yielding six differential metabolite sets. Six pairs of differentially expressed genes and differential metabolites were selected for correlation analysis. The six pairs of data for analysis were: BYR6_vs_CK, BAR3_vs_CK, MIX36_vs_CK, BYR6_vs_MIX36, BAR3_vs_MIX36, and BAR3_vs_BYR6.

### 5.6 Fluorescent quantitative PCR

Total RNA was extracted from the *C. equisetifolia* seedling tissues and was reverse-transcribed into cDNA. Ten genes identified through transcriptomic sequencing as differentially expressed between various treatment groups were randomly selected for qRT-PCR validation. The system was prepared with a total volume of 20 μL and a cDNA volume of 2 μL. Once mixed, the samples were placed in the ABI7300 fluorescence quantitative PCR instrument (Applied Biosystems, USA) for quantitative analysis. The reaction conditions were set to preheat at 95°C for 5 min, followed by 40 cycles of amplification (denature at 95°C for 5 s, anneal at 55°C for 30 s, extend at 72°C for 40 s). The target genes included 4-coumarate-ligase2 (4CL), Chalcone synthase (CHS), Cytochrome P450 family 75A (CYP75A), Hydroxycinnamoyl transferase (HCT), Lipoxygenase isoform 1(LOX2S), Dihydroflavonol 4-reductase(DFR), Auxin-repressed kDa-like, Light-regulated-like, Catalase and Metallothionein1, and EF1α was used as the internal reference, the 2^-ΔΔCт^ analysis was performed, and the primer sequences were provided in **Supplementary Table 6**.

### 5.7 Statistical analysis

Data were analyzed using IBM SPSS20.0 statistical software. A one-way analysis of variance (ANOVA) was performed for comparisons among multiple groups, and *P* < 0.05 was considered statistically significant. GraphPad Prism 7 software was utilized to plot and analyze the Pearson correlations between the transcriptomic sequencing data and qRT-PCR data.

## Data Availability

The datasets presented in this study can be found in online repositories. The names of the repository/repositories and accession number(s) can be found in the article/**Supplementary Material**.
